# LIFE RESTORATION AND CARE PREPARATION AMONG OLDER PARENTS WHO LOST THEIR ONLY ADULT CHILD IN SHANGHAI

**DOI:** 10.1093/geroni/igz038.1019

**Published:** 2019-11-08

**Authors:** Lin Chen, Yajun Song, Yixin You

**Affiliations:** 1 Fudan University, Shanghai, China; 2 East China University of Science and Technology, Shanghai, China, China

## Abstract

The combination of aging and losing an only adult child challenges an increasing number of older adults in China. Current literature primarily focuses on older parents’ bereavement but seldom examines how they restore their lives. Guided by the Dual Process Model, this study explores how older parents who lost their only adult child restore their lives and prepare for future care in Shanghai. Twenty-four older adults were purposively sampled and participated in face-to-face, in-depth interviews. The findings suggest that participants tried to restore their lives by rationalizing grief and expanding their social networks. Despite their losses, participants remained in favor of family caregiving and reluctantly prepared for future care. They showed ambivalent attitudes toward current government support while proposing their preferred services. This study informs social work practice to incorporate caregiving plan services and emotional support for this vulnerable group.

